# Predictors of preterm neonatal mortality in the neonatal intensive care unit at a tertiary medical institution in Ethiopia

**DOI:** 10.3389/fped.2025.1414127

**Published:** 2025-09-19

**Authors:** Tihun Feleke, Gudeta Kaweti

**Affiliations:** ^1^ Department of Public Health, Hawassa College of Health Sciences, Hawassa, Ethiopia; ^2^ School of Social and Population Health, Yirgalem Hospital Medical College, Yirgalem, Ethiopia

**Keywords:** preterm neonatal mortality, Sidama, Ethiopia, Hawassa, Hawassa University

## Abstract

**Background:**

Premature death is a serious health concern in developing countries, including Ethiopia.

**Methods:**

A retrospective cohort study was conducted in Hawassa University Comprehensive Specialized Hospital from 9 May 2019 to 22 April 2021. A total of 723 preterm neonates were enrolled in this study. The Kaplan–Meier survival curve was used to calculate the survival rate. The Cox proportional hazard ratio was used to evaluate the relationship between the dependent and independent variables. A 95% confidence level was used to check for significance.

**Results:**

Preterm neonatal mortality accounted for 33.3% of neonatal admissions. Early neonatal sepsis [adjusted hazard ratio (AHR) = 1.34; 95% CI: 1.003, 1.79], a 5-min Apgar score of less than 7 (AHR = 1.73; 95% CI: 1.17, 2.55), perinatal asphyxia (AHR = 2.25; 95% CI: 1.67, 3.02), and recent multiple pregnancies (AHR = 1.66; 95% CI: 1.22, 2.26) were predictors of preterm neonatal mortality.

**Conclusion:**

Early breastfeeding, prevention and early treatment of perinatal hypoxia and neonatal infections, identification, and monitoring of multiple pregnancies could help to reduce preterm neonatal mortality.

## Introduction

The World Health Organization targeted a 50% decrease in preterm fatalities by 2025 in nations with neonatal mortality rates of more than 5 per 1,000 live births in 2012 (1). Moreover, lowering the burden of premature mortality is crucial for reaching Sustainable Development Goal 3 (2). Stabilization, early detection, and treatment of infections; the prevention of hypothermia; early introduction of breast milk; and more intensive neonatal care are all viable and affordable measures that can prevent more than three-quarters of these premature neonatal deaths (3).

Every year, more than 1 million out of every 15 million babies are born preterm (4). This is a significant public health problem across the world because of the associated morbidity and mortality (5). It is a single, direct leading cause of death, accounting for approximately 18% of all deaths in children under 5 and 35% of all neonatal deaths worldwide (6). One-third of all preterm neonatal deaths occur within the first day after birth, and close to three-quarters of preterm neonatal deaths occur within the first week of life worldwide (1, 4).

The child mortality rate in Africa declined by 42% in 2012 compared to a 60%–72% decline in other regions of the world (7). This mortality rate is higher within the first week of life. In Nigeria, 73% of deaths occurred within the first week of life (8). Preterm neonates born in sub-Saharan Africa are 10 times more likely to die compared to developed countries (4, 9). Over 90% of extremely preterm babies born in low-income countries die within the first few days of life, while less than 10% die in high-income countries (1, 9). In East Africa, the survival rate was 94.6% and 52.6% for preterm neonates born at or after 34 weeks of gestational age and less than 34 weeks of gestational age, respectively (10).

In Ethiopia, the preterm mortality rate is high, ranking 10th in the world in 2010 (7). According to a study conducted in northern Ethiopia, the preterm neonatal death rate was 37% (11). Another study conducted in northern Ethiopia revealed that 11.4% of admitted preterm neonates died in the first 24 h and 85.27% died in the first 7 days (12).

Preterm neonates who are born before reaching maturity are fragile, small, weigh less than a full-term infant, and face a variety of physiological handicaps (13). A lack of feasible, cost-effective care, such as warmth, breastfeeding support, and basic care for infections and breathing difficulties, presents a challenge to their survival (8). Those who survive often face a lifetime of ill-health, including disability, learning difficulties, visual and hearing problems, psychological crises, financial hardships, and economic burden for the families and broader society due to their long-term complex healthcare needs (4, 10, 12).

Despite various efforts, the mortality rate due to complications related to preterm births has still not been reduced as expected (14). The purpose of this study was to fill the gap in knowledge of preterm neonatal mortality, provide data for further studies, and indicate further interventional measures. Conducting this study is helpful as there is limited data on the status of preterm neonatal mortality and its associated factors, especially in southern Ethiopia. The findings will inform public health authorities about the recent burden due to preterm neonatal mortality and its contributing factors, allowing them to tailor interventions to solve the identified problems.

## Methods

### Study’s setting, design, and period

This study was carried out at Hawassa University Comprehensive Specialized Hospital (HU-CSH), a neonatal intensive care unit (NICU)-affiliated tertiary-level medical institution in Ethiopia that is 275 km south of Addis Ababa. It has an average of 350–400 and 1,000–1,500 annual admissions for premature and neonatal patients, respectively. A facility-based retrospective cohort analysis was carried out on data from hospitalized patients between 9 May 2019 and 22 April 2021.

### Inclusion and exclusion criteria

#### Inclusion criteria

All preterm neonates hospitalized in the NICU-affiliated tertiary hospital were included.

#### Exclusion criteria

At the time of data collection, records of preterm neonates with missing information, such as date of birth, length of stay, premature outcome, and missing patient cards, were excluded.

### Sample size

During the study period, all hospitalized preterm neonates were considered. As a result, no sample size was used.

#### Data collection tool

After a careful analysis of the literature and any necessary adjustments, a standardized data abstraction checklist was created and used for data collection. Training was provided to four registered nurses before data collection. The data collection period was from 9 to 30 May 2021.

#### Sampling procedure

Each preterm neonate's medical information was obtained using their record number from the NICU’s registration book. Data collection (both for baseline and follow-up records) was conducted after the chart was reviewed. All research participants' records were chosen based on the eligibility criteria.

#### Measurement and variables

The follow-up began on the initial day of NICU admission and ended on the date of death or discharge. Preterm neonatal death was the outcome variable. The data acquired from the chart of the mothers of each preterm neonate who was eligible for the trial were used to determine the outcome. Sociodemographic characteristics, maternal obstetric-associated factors, maternal medical-related factors, and neonatal factors were the independent variables.

### Data quality management

The use of a standard data abstraction process, using trained BSC nurses for data collection, and daily reviews by primary investigators to guarantee consistency and completeness all contributed to the data's high quality. By choosing charts at random, data integrity was maintained.

### Statistical analysis

EpiData version 3.1 was used to enter and clean the data, which were then exported and analyzed using STATA version 14. Each participant's outcome was classified as either discharged or death. The period between the dates of delivery and the date of death, discharge, or the end of the research was used to calculate the survival status of the preterm neonates. The Kaplan–Meier method was used to determine the mean survival time and cumulative probability of survival, and log-rank tests were employed to compare the survival curves of the groups following admission to the NICU. A multivariable Cox proportional hazards regression model with a 95% confidence interval was fitted to variables with *p*-values of 0.25 in the bivariate analysis, and a *p*-value of 0.05 was judged to be statistically significant. The model's goodness-of-fit was evaluated using Cox–Snell residuals. The assumption of the Cox proportional hazard regression model was also tested using the Schoenfeld residual test, and variables with a *p*-value greater than 0.05 were evaluated to have met the assumption.

## Results

### Sociodemographic characteristics of the preterm neonates and their mothers

**Figure 1:**
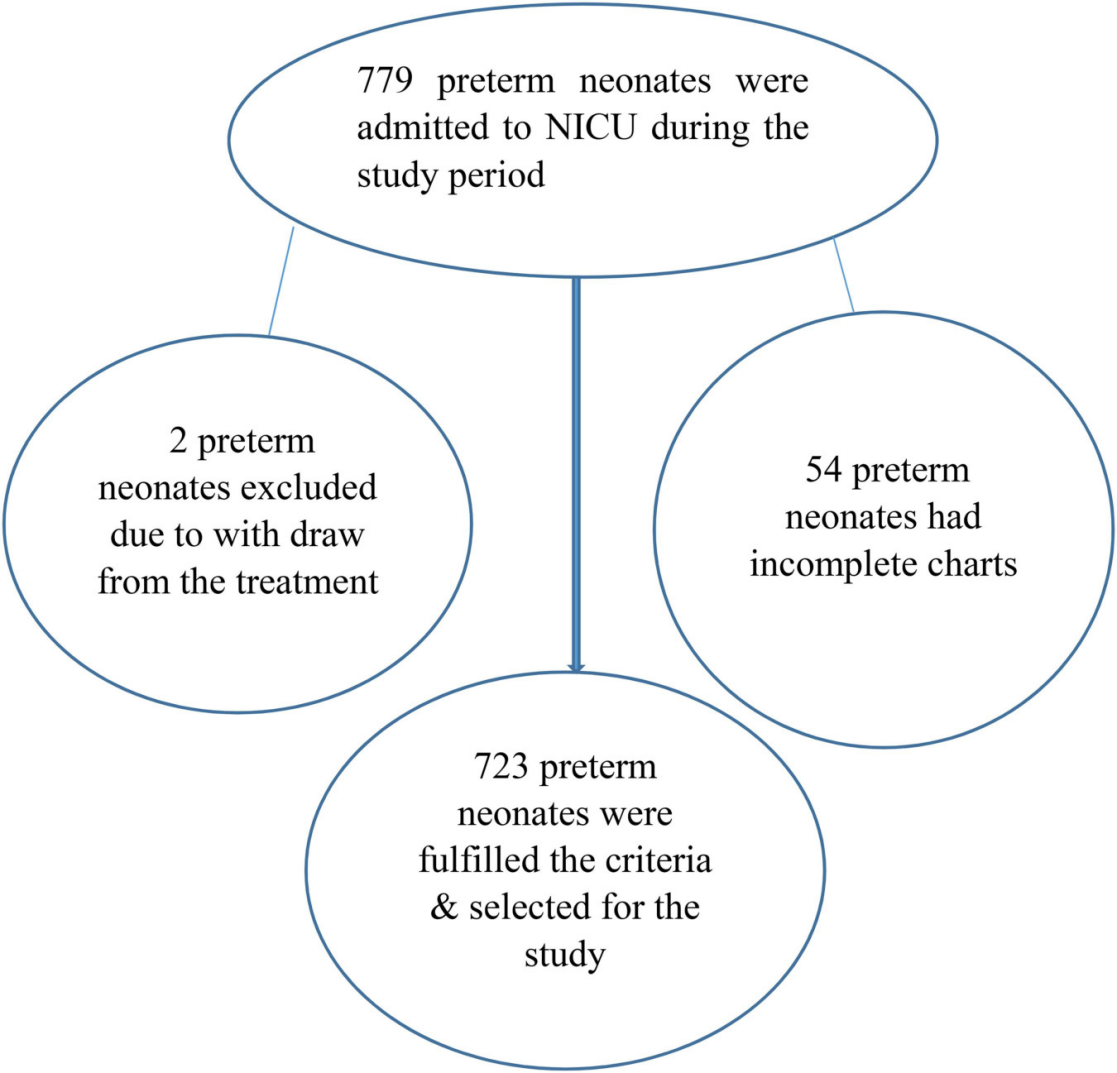
Flow chart of the recruitment of preterm neonates from a tertiary-level medical institution in Ethiopia from May 2019 to April 2021.

Of the 779 charts reviewed, 723 (92.8%) met the enrollment criteria and were included (Figure 1). Among the 723 preterm neonates, more than half (51.87%) were females and approximately three-fifths (60.7%) resided in urban areas. The mean length of hospital stay was 10.4 days (95% CI: 9.8, 10.9) and the median was 11 days (95% CI: 9.2, 12.81). In total, 241 (33.3%) preterm neonates died, among whom 126 were female (52.28%). Moreover, 12.2% of the mothers were aged 20 years or younger and 61% of them were from urban areas (Table 1).

**Table 1:** Sociodemographic characteristics of the preterm neonates and their mothers who were admitted to a neonatal intensive care unit of a tertiary-level medical institution in Ethiopia in 2021 (*n* = 723).

Variable	Frequency	Percentage
Sex		
Female	375	51.9
Male	348	48.1
Residence		
Urban	438	60.6
Rural	285	39.4
Neonatal age at admission		
<24 h	567	78.4
1–7 days	139	19.2
>7	17	2.4
Maternal age		
≤20	88	12.2
20–34	547	75.7
>34	88	12.2
Estimated gestational age		
28–31	378	52.28
32–36	345	47.72
Place of delivery		
HU-CSH	397	54.9
Other health institutions	316	43.7
Home	10	1.4
Length of hospital stay		
<24 h	30	4.1
1–7 days	275	38
≥7 days	418	57.8

Note: Mean and median length of stay were 10.4 (95% CI: 9.8, 10.9) and 11 (95% CI: 9.2, 12.81), respectively.

#### Obstetric and medical-related characteristics of the mothers

The mean age of the mothers was 27 ± 5.138 years. Approximately half of the mothers (49%) had a gravidity of less than or equal to two, and the majority of the mothers (64.5%) had a parity of less than or equal to two. Moreover, 10 (1.4%) of the participants gave birth at home, accounting for 2% of premature infant deaths. Finally, 42.2%, 56.2%, and 94.5% of the mothers underwent a cesarean section, had spontaneous vaginal delivery, and attended antenatal care (ANC) follow-up, respectively (Table 2).

**Table 2:** Medical and obstetric-related characteristics of the mothers of the neonates who were admitted to the neonatal intensive care unit of a tertiary-level medical institution in Ethiopia in 2021 (*n* = 723).

Variable	Frequency	Percent
Mode of delivery		
Spontaneous VD	406	56.2
Cesarean section	305	42.2
Instrumental	12	1.7
Multiple pregnancies		
Yes	79	10.9
No	644	89.1
Parity		
≤2	466	64.5
>2	257	35.5
Gravidity		
≤2	354	49
>2	369	51
At least one ANC visit		
Yes	684	94.6
No	39	5.4
PROM		
Yes	96	13.9
No	627	86.1
Placental abruption		
Yes	42	5.8
No	681	94.2
Anemia		
Yes	21	2.9
No	702	97.1
Hypertension		
Yes	43	5.9
No	680	94.1
Other		
Yes	29	4.01
No	694	95.99

PROM, premature rupture of membranes; VD, vaginal delivery.

### Incidence of death among preterm neonates

A cohort of 723 premature neonates was retrospectively followed from admission to 28 days of age. The total observation time of the study participants was 7,501 person-days. The overall mortality rate was 32.12% (95% CI: 28.31, 36.45). The majority of the preterm neonates, 393 (81.5%), were discharged home in good health, 84 (17.2%) were discharged against medical advice, and 6 (1.3%) were referred. All the preterm babies who weighed less than 1,000 g at birth died.

### Factors associated with preterm neonatal mortality

In the final model, breastfeeding, early neonatal sepsis, 5-min Apgar score of less than 7, perinatal asphyxia, and multiple pregnancies were found to be significant predictors of mortality in preterm neonates.

Early initiation of breastfeeding in preterm neonates reduced the risk of death by 57% compared to a lack of early initiation of breastfeeding [adjusted hazard ratio, AHR: 0.43 (95% CI: 0.29, 0.62)]. The risk of death for preterm neonates whose mother attended an ANC follow-up was reduced by 48% compared to their counterparts [AHR: 0.52 (0.30, 0.85)]. Mortality among preterm neonates diagnosed with early neonatal sepsis was 1.34 times more likely compared to those neonates who were not diagnosed with early neonatal sepsis at admission [AHR: 1.34 (95% CI: 1.003, 1.79)]. Preterm neonates who had an estimated gestational age of 28–31 weeks were 1.44 times more likely to die than those who were between 32 and 36 weeks of estimated GA (AHR = 1.44: 95% CI: (1.02, 2.03). The likelihood of mortality among preterm neonates who had a 5-min Apgar score of less than 7 increased by 73% compared to their counterparts [AHR: 1.73 (95% CI: 1.17, 2.55)]. The likelihood of mortality among preterm neonates diagnosed with perinatal asphyxia was 2.25 times higher compared to preterm neonates who were not diagnosed with perinatal asphyxia at the time of admission [AHR: 2.25 (95% CI: 1.67, 3.02)]. The likelihood of mortality among preterm neonates whose mother had recent multiple pregnancies was 1.66 times higher compared to those preterm neonates whose mothers had no recent history of multiple pregnancies [AHR: 1.66 (1.22, 2.26)]. Of the 723 neonates, breastfeeding history was available for 674, and missing data for 49 cases led to their exclusion from the breastfeeding analysis (Table 3).

**Table 3:** Results of the bivariate and multivariable Cox regression analyses for the preterm neonates admitted to the NICU of a tertiary-level medical institution in Ethiopia in 2021 (*n* = 723).

Variable	Died (%)	Discharged (%)	CHR (95% CI)	AHR (95% CI)
Birth weight				
<1,500 g	20 (100)	0 (0)	2.6 (1.26, 5.34)	2.49 (0.58, 2.75)
1,500–2,500 g	131 (25.8)	376 (74.2)	1.08 (0.53, 2.21)	1.06 (0.51, 2.23)
>2,500 g	8 (22.9)	27 (77.1)	1	1
1-min Apgar score				
<7	228 (35.3)	417 (64.7)	2.21 (1.27,3.87)	1.07 (0.56, 2.07)
≥7	13 (16.7)	65 (83.3)	1	1
5-min Apgar score				
<7	197 (44.1)	250 (55.9)	3.0 (2.17, 4.17)	1.91 (1.30,2.81)**
≥7	44 (15.9)	232 (84.1)	1	1
Breastfeeding				
Yes	39 (15.3)	216 (84.7)	0.29 (0.21, 0.41)	0.41 (0.28, 0.59)***
No	179 (42.7)	240 (57.32)	1	1
Neonatal sepsis				
Yes	84 (42.4)	114 (57.6)	1.89 (1.47, 2.43)	1.37 (1.03, 1.83)*
No	157 (29.9)	368 (70.1)	1	1
Perinatal asphyxia				
Yes	74 (69.8)	32 (30.2)	3.72 (2.82, 4.91)	2.64 (1.93, 3.61)***
No	167 (27.1)	450 (72.9)	1	1
Hypothermia				
Yes	176 (39.7)	267 (60.3)	1.86 (1.40, 2.47)	1.27 (0.92,1.77)
No	65 (23.2)	215 (76.8)	1	1
Multiple pregnancies				
Yes	24 (30.4)	55 (69.6)	2.15 (1.63, 2.84)	1.82 (1.35, 2.46)***
No	217 (33.7)	427 (66.3)	1	1
Maternal anemia				
Yes	13 (61.9)	8 (38.1)	1.97 (1.12,3.45)	1.53 (0.79, 2.95)
No	8	474 (67.5)	1	1
ANC follow-up				
Yes	224 (32.7)	460 (67.3)	0.65 (0.34, 1.03)	0.52 (0.30, 0.85)*
No	17 (43.6)	22 (56.4)	1	1
EGA (weeks)				
28–31	89 (23.5)	289 (76.5)	2.01 (1.55,2.6)	1.44 (1.02, 2.03)*
32–36	152 (44.1)	193 (55.9)	1	1
Placental abruption				
Yes	218 (32.0)	463 (68.0)	1.96 (1.28, 3.01)	1.29 (0.75, 2.21)
No	23 (54.8)	19 (45.2)	1	1

* *p* < 0.05.

** *p* < 0.01.

*** *p* < 0.001.

The Schoenfeld residuals and global test were >0.05 (0.27); a relatively safe population from a scientific point of view was used as the reference category. Important variables, including birth location and place of residence, were not eligible for the final model.

EGA, estimated gestational age.

### Survival and 5-min Apgar score

**Figure 2:**
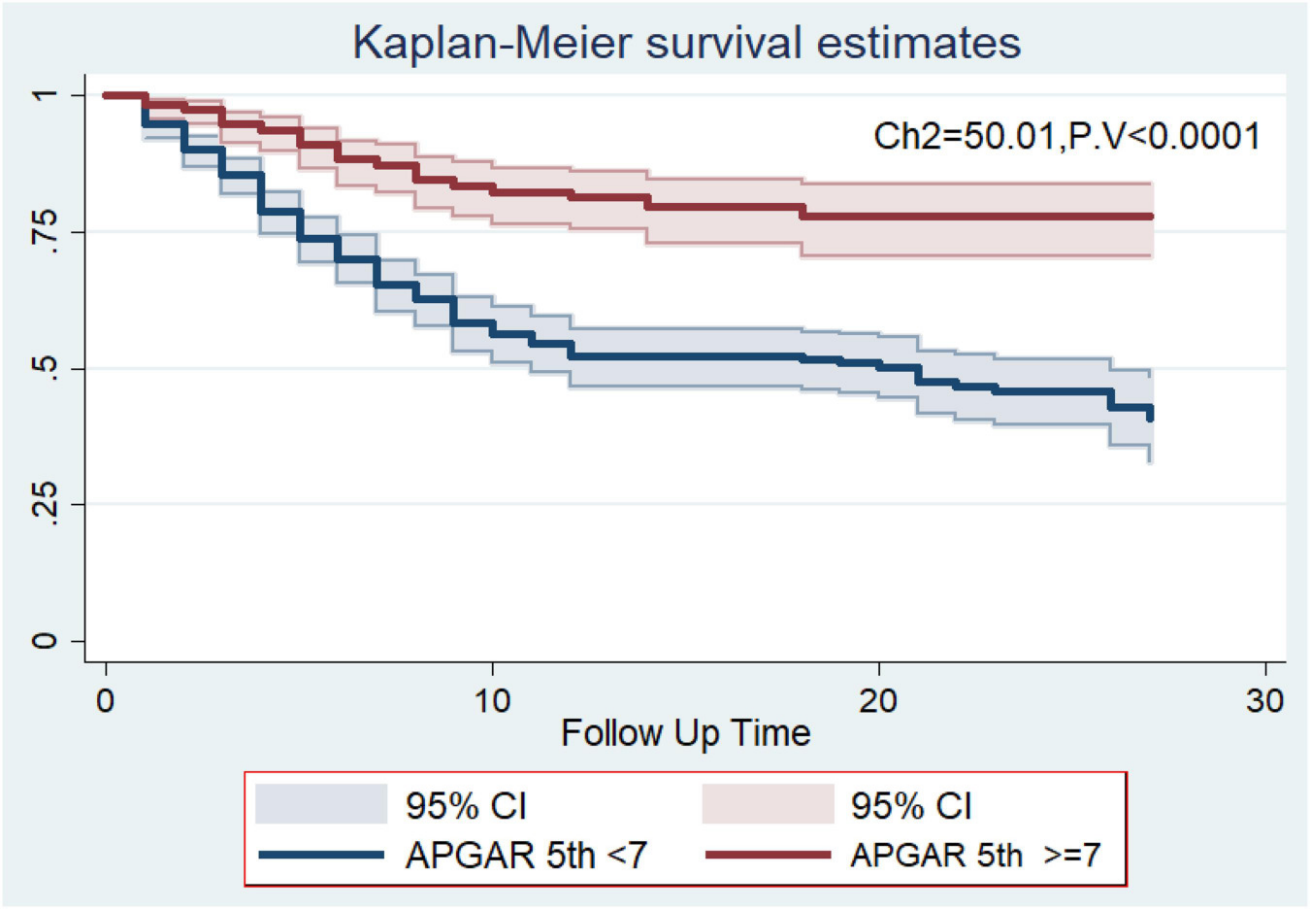
The Kaplan–Meier survival curves compare the survival time of preterm neonates with different 5-min Apgar scores at a tertiary-level medical institution in Ethiopia from May 2019 to April 2021.

In this study, the overall survival rate of preterm neonates with a 5-min Apgar score of less than 7 was 40.8%, whereas the survival rate of the preterm neonates with a 5-min Apgar score of >7 was 77.8% at the end of the follow-up period (*p*-value = 0.001). The median survival time for preterm neonates with a 5-min Apgar score of less than 7 was 21 days (95% CI: 14.1, 27.9) (Figure 2).

## Discussion

The mortality rate among the preterm neonates during the study period was 33.3%. This finding was comparable with studies conducted in Jimma (34.9%) (15) and at Felegehiwot Hospital (34.1%) (16) in Ethiopia and findings from other developing countries, such as Uganda (31.6%) (5). However, it was slightly higher than the findings from studies conducted at the University of Gondar (25.2%) (12), at a tertiary care hospital in Addis Ababa (23.2%) (17); the findings from studies conducted in other developing countries such as Nigeria (27.69%) (18), South Africa (Johannesburg Hospital, 26.5%), and Cameroon (15.7%) (19); and the findings of studies conducted in developed countries such as Iran (27.4%) (20). However, it was lower than studies conducted in a low-income country (37.5%) (21) and urban Pakistan (47.3%) (22). The reasons for these disparities could be due to variation in the study settings and the quality of the services provided in these settings, including differences in NICU standards, the quantity and quality of trained health professionals, the health service utilization practices of the community, and treatment modalities.

The proportion of preterm neonates with a 5-min Apgar score less than 7 was 61.8%, which is high. This could be due to insufficient equipment, space shortage, high workloads, and a knowledge gap, which are actually the usual problems in developing countries. Of the preterm neonates enrolled in this study, 61.3% were hypothermic, of which 39.7% died. A high workload and insufficient equipment may be the cause of this mortality rate. Simple equipment, such as warm blankets, hats, and plastic wraps, and the implementation of the Neonatal Resuscitation algorithm protocols would reduce instances of neonatal hypothermia.

Preterm neonates whose mother attended an ANC follow-up were 48% less likely to die when compared to those preterm neonates whose mother attended no ANC follow-up [AHR, 0.52 (0.30, 0.85)] (*p* < 0.05). This finding was consistent with studies conducted at University of Gondar Specialized Hospital (12) and Felegehiwot Hospital (16).

Preterm neonates who had a 5-min Apgar score of less than 7 were 91% more likely to die compared to their counterparts [AHR: 1.91 (95% CI: 1.30, 2.81)] (*p* < 0.01). This finding was supported by the findings of studies conducted in China (23), Brazil (24), Iran (20), Cameroon (19), Ethiopia (12, 25, 26), and Ghana (3). A shortage of space for neonatal intensive care services, a shortage of resuscitation equipment, missed diagnoses of preterm neonate complications, and a delay in the identification and management of newborn complications may be among the reasons for this finding.

The likelihood of mortality among preterm neonates with perinatal asphyxia at the time of admission was approximately 3 times higher than that among the preterm neonates who had no perinatal asphyxia at the time of admission [AHR = 2.64 (95% CI: 1.93, 3.61)] (*p* < 0.001). This finding was in line with the findings of studies conducted in a tertiary hospital in Addis Ababa (17) and those conducted at Mizan Tepi Hospital (25) and Felegehiwot Hospital in Bahir Dar (16). The high rate of mortality among the preterm neonates with perinatal asphyxia in this study, 74 (69.8%) deaths compared to 167 (27.1%) among the preterm neonates without birth asphyxia, is of particular interest. This is because it is a preventable cause of neonatal death and the high mortality rate in the neonatal care unit could be related to the inadequate service quality.

The early initiation of breastfeeding decreased the risk of preterm neonatal mortality by 59% [AHR = 0.41: 95% CI: (0.28, 0.59)] (*p* < 0.001). This finding was supported by a previous study conducted in northwest Ethiopia (27). There is scientific evidence that the initiation of breastfeeding and/or feeding with breast milk may provide protection against various diseases, as breast milk has antibacterial, immunological, and other properties that can increase bactericidal enzymes, complements, and macrophages (28).

The risk of mortality among preterm neonates who had a gestational age of 28–31 weeks at the time of admission was 44% higher compared to the preterm neonates who had a gestational age between 32 and 36 weeks at admission [AHR = 1.44: 95% CI (1.02, 2.03)] (*P* < 0.05). This finding was supported by studies conducted in Jimma (15), Gondar (12), Mizan Tepi (25), and Iran (20). This may be due to the immaturity of different organ systems in preterm neonates, which leads to an inability to resist the external environment.

Preterm neonates diagnosed with early neonatal sepsis during admission had a 37% increased risk of mortality compared to the preterm neonates with no early neonatal sepsis [AHR = 1.37 (95% CI: 1.03, 1.83)] (*p* < 0.05). This finding was supported by studies conducted in Gondar (12), Mizan Tepi (25), Jimma (15), and Iran (20). Preterm neonates born with immature body self-defense mechanisms and other procedure-related factors could also have contributed to the existence of these differences.

Recent multiple pregnancies increased the risk of death for preterm neonates by approximately 2 times compared to a singleton pregnancy [AHR: 1.82 (1.35, 2.46)] (*p* < 0.01). This finding was supported by a study conducted in Iran (20).

## Strengths

The study design was a retrospective cohort study that enabled a comparison between preterm neonates who died and those who were discharged. This provides an important road map and insight for those researchers who have an interest in conducting research utilizing a better study design (for instance, a prospective cohort study). The result of the study was representative of the preterm neonates born at Hawassa University Comprehensive Specialized Hospital. The use of a multivariate Cox proportional regression model provided the maximum chance of controlling for any possible confounders.

## Limitations

The findings of this study should be interpreted with consideration of its limitations. There was a high chance of missing some important predictor variables as the data were collected from a secondary source. Generalization of the study’s findings to other health institutions in the country is impossible as this study was conducted in only one hospital (i.e., Hawassa University Comprehensive Specialized Hospital). There was a high chance of selection bias due to the exclusion of incomplete records, which may have led to under- or overestimation of the preterm neonatal mortality. Some non institutional deliveries may affect the result.

Breastfeeding history was available for 674 of the 723 neonates included in our retrospective cohort study, while the remaining 49 cases had missing breastfeeding data due to incomplete records. This is a known limitation of retrospective data collection. These cases were excluded from the analyses involving breastfeeding history to maintain methodological rigor. We acknowledge this limitation and have transparently reported it since we believe that the sample size remained sufficient to support valid and reliable conclusions.

## Conclusion and recommendations

The magnitude of preterm neonatal mortality at this tertiary-level medical institution was high (33.3%). The early initiation of breastfeeding was protective against preterm neonatal mortality, while having a 5-min Apgar score of less than 7, prenatal asphyxia, early neonatal sepsis, and recent multiple pregnancies were factors associated with preterm neonatal mortality. Having enough skilled manpower, improving the quality of care at ANC follow-ups for mothers, equipping the NICU with adequate infrastructure, and providing special care for preterm neonates could mitigate complications related to preterm birth.

Various maternal and neonatal risk factors were identified, indicating a need for stakeholders to enhance efforts toward the prevention of preterm-birth-associated complications. Furthermore, it would be helpful to optimize the facility-based continuum of care. We recommend that research with a rigorous study design be conducted with a broader scope that focuses on both facilities and communities to provide a more holistic understanding of the root causes of preterm neonatal mortality and inform future interventions.

## Data Availability

The original contributions presented in the study are included in the article/Supplementary Material, further inquiries can be directed to the corresponding author.
